# Machine learning prediction of postoperative major adverse cardiovascular events in geriatric patients: a prospective cohort study

**DOI:** 10.1186/s12871-022-01827-x

**Published:** 2022-09-10

**Authors:** Xiran Peng, Tao Zhu, Tong Wang, Fengjun Wang, Ke Li, Xuechao Hao

**Affiliations:** 1 Department of Anesthesiology, National Clinical Research Center for Geriatrics, West China Hospital, Sichuan University, PO Box 610041, Chengdu, China; 2 The Research Units of West China (2018RU012) Chinese Academy of Medical Sciences, West China Hospital, Sichuan University, Chengdu, China; 3 Center of Statistical Research, School of Statistics, Southwestern University of Finance and Economics, Chengdu, China; 4 Joint Lab of Data Science and Business Intelligence, School of Statistics, Southwestern University of Finance and Economics, PO Box 611130, Chengdu, China

**Keywords:** Postoperative major adverse cardiovascular events, Risk assessment, Geriatric assessment, Machine learning, Electronic health records

## Abstract

**Background:**

Postoperative major adverse cardiovascular events (MACEs) account for more than one-third of perioperative deaths. Geriatric patients are more vulnerable to postoperative MACEs than younger patients. Identifying high-risk patients in advance can help with clinical decision making and improve prognosis. This study aimed to develop a machine learning model for the preoperative prediction of postoperative MACEs in geriatric patients.

**Methods:**

We collected patients’ clinical data and laboratory tests prospectively. All patients over 65 years who underwent surgeries in West China Hospital of Sichuan University from June 25, 2019 to June 29, 2020 were included. Models based on extreme gradient boosting (XGB), gradient boosting machine, random forest, support vector machine, and Elastic Net logistic regression were trained. The models’ performance was compared according to area under the precision-recall curve (AUPRC), area under the receiver operating characteristic curve (AUROC) and Brier score. To minimize the influence of clinical intervention, we trained the model based on undersampling set. Variables with little contribution were excluded to simplify the model for ensuring the ease of use in clinical settings.

**Results:**

We enrolled 5705 geriatric patients into the final dataset. Of those patients, 171 (3.0%) developed postoperative MACEs within 30 days after surgery. The XGB model outperformed other machine learning models with AUPRC of 0.404(95% confidence interval [CI]: 0.219–0.589), AUROC of 0.870(95%CI: 0.786–0.938) and Brier score of 0.024(95% CI: 0.016–0.032). Model trained on undersampling set showed improved performance with AUPRC of 0.511(95% CI: 0.344–0.667, *p* < 0.001), AUROC of 0.912(95% CI: 0.847–0.962, *p* < 0.001) and Brier score of 0.020 (95% CI: 0.013–0.028, *p* < 0.001). After removing variables with little contribution, the undersampling model showed comparable predictive accuracy with AUPRC of 0.507(95% CI: 0.338–0.669, *p* = 0.36), AUROC of 0.896(95%CI: 0.826–0.953, *p* < 0.001) and Brier score of 0.020(95% CI: 0.013–0.028, *p* = 0.20).

**Conclusions:**

In this prospective study, we developed machine learning models for preoperative prediction of postoperative MACEs in geriatric patients. The XGB model showed the best performance. Undersampling method achieved further improvement of model performance.

**Trial registration:**

The protocol of this study was registered at www.chictr.org.cn (15/08/2019, ChiCTR1900025160)

**Supplementary Information:**

The online version contains supplementary material available at 10.1186/s12871-022-01827-x.

## Background

More than 300 million surgeries are performed worldwide annually [1]. About one-third of elective surgeries are performed on adults aged over 65 years [2]. Although surgery has the potential advantages of improving quality of life and prolonging the patient’s lifespan, perioperative complications may offset the benefits [3].

Postoperative major adverse cardiovascular events (MACEs) account for more than one-third of perioperative deaths [4, 5]. Geriatric patients are more likely to develop postoperative MACEs because of age-related threats such as comorbidity, polypharmacy, and frailty [6].

Early identification of high-risk patients would allow for advance interventions and facilitate prevention of postoperative MACEs. The Revised Cardiac Risk Index (RCRI) and the Gupta Myocardial Infarction or Cardiac Arrest (MICA) are the most widely used tools for evaluating the probability of postoperative MACEs. Considering that these tools were developed several years ago, they have some limitations.

First, RCRI and MICA both tend to underestimate the risk of postoperative MACEs [7], especially in the high-risk group [8]. Compared with the general population, geriatric patients have a much higher risk of MACEs [9]. RCRI and MICA show moderate performance when applied to the geriatric population, often underestimating the real cardiac risk [9]. Second, RCRI and MICA were both developed based on logistic regression. Constraints in the logistic regression confined these models to a small group of variables, which may overlook potentially valid predictors [10].

Comprehensive clinical information coupled with laboratory tests generate a large amount of data. Machine learning is an optimal choice for analyzing complex datasets [11]. Recent studies using machine learning methods to predict the risk of postoperative MACEs have often focused on specific types of surgery [12, 13], which limits the applicability of such models to a wider range of surgeries. Moreover, none of those tools were developed specifically for geriatric patients.

In this study, we aimed to use prospectively collected data to develop a machine learning model for preoperative prediction of postoperative MACEs in geriatric patients. We hypothesized that this machine learning model could improve the prediction of postoperative MACEs in geriatric patients.

## Methods

### Data source

For this study, we created a longitudinal cohort and collected data prospectively at West China Hospital of Sichuan University, a 4000-bed tertiary academic hospital in China. The protocol of this study was approved by Committee of Ethics from West China Hospital of Sichuan University (2019–473) with waiver of informed consent, and registered at www.chictr.org.cn (15/08/2019, ChiCTR1900025160). We designed our own preoperative interview sheet to capture related information. Trained residents used this sheet to interview patients and collect data on the day before surgery. The attending physician and resident re-checked the collected information before surgery. If any omission or error existed, the clinician made the addition or correction. Preoperative laboratory tests were automatically retrieved from the Laboratory Information System. Preoperative data involved patients’ demographic information, preoperative vital signs, comorbidities, laboratory tests, and surgical details. Supplementary table S1 shows the 121 variables included in our study. Instead of simply categorizing comorbidities according to the presence or absence of each disease, we classified some diseases by severity. For example, hypertension was categorized according to blood pressure level. All laboratory tests were done within 7 days before surgery. If a patient had more than one result for the same test, we chose the most recent result before surgery. We enrolled all patients aged over 65 years who underwent surgeries from June 25, 2019 to June 29, 2020. Patients were excluded if they (1) had active symptoms of MACEs before surgery; (2) lost to follow-up.

### Postoperative follow-up

To ascertain the presence of postoperative MACEs, we conducted prospective follow-up with the patients. Research personnel followed up with patients at different time points after surgery, including 24 h after surgery, before hospital discharge, and the 30^th^ day after surgery. If a patient developed postoperative MACEs, we continually stayed in contact with the patient until recovery or death. Throughout each patient’s hospital stay, research personnel conducted bedside follow-up visits; after hospital discharge, patients were contacted via phone call.

### Outcome definition

The outcome was postoperative MACEs within 30 days after surgery. MACEs included myocardial ischemia, cardiac arrest, high-risk arrhythmia, heart failure, and stroke. Postoperative outcome data were collected from our electronic follow-up system.

Myocardial ischemia was defined by the presence of one of the following: (1) electrocardiogram showing signs of myocardial infarction (any one of the following): (a) ST segment elevation > 1 mm in two or more adjacent leads, (b) new left bundle branch, or (c) new Q-wave in two or more adjacent leads; or (2) new troponin elevation beyond 3 times the upper limit of the reference value in patients with suspected myocardial infarction.

Cardiac arrest was defined as “loss of cardiac impulse or the presence of an abnormal cardiac rhythm that leads to complete unconsciousness requiring basic or advanced life support.” This definition included malignant ventricular or supraventricular arrhythmias, pulseless electrical activity, and asystole.

High-risk arrhythmia referred to ventricular fibrillation. Heart failure was defined as the appearance of any one of the following: dyspnea, palpitation, or chest pressure after exercise; pulmonary edema; physical examination showing bilateral rales; or chest radiograph showing butterfly sign. Stroke was defined as “cerebrovascular events caused by intracranial vascular rupture, thrombosis, or embolism.”

### Data preprocessing and model development

All variables were presented as continuous or categorical variables. Missing values were interpolated before modeling. Continuous variables missing in more than 10% of cases and categorical variables were imputed by − 99, which regarded missing values as a separate group [14]. Continuous variables missing in fewer than 10% of cases were imputed using the k-nearest neighbor classification algorithm [15]. This nearest-neighbor based technique is a standard missing value imputation method, which predicts the missing values through selecting a group of patients with corresponding values in similar condition to the patient with missing values [15].

We randomly selected 80% of all observations for training, leaving 20% for testing. The classification methods on which models were based included extreme gradient boosting (XGB)[16], gradient boosting machine (GBM)[17], random forest (RF) [18], support vector machine (SVM) and Elastic Net logistic regression[19]. Elastic Net logistic regression and SVM are based on distance measurement, which indicates the need of standardization of features. We rescaled the value between 0 and 1 using Min–max normalization.

The number of patients without postoperative MACEs was much higher than the number of patients with postoperative MACEs, which leading to extreme class imbalance. This issue was overcome through setting different sample weights. In RF, SVM and Elastic Net logistic regression, the hyperparameter “class_weight” was set to “balanced” to automatically increase the weight of positive sample. In GBM, the hyperparameter “sample_weight” was used to decrease the weight of negative sample and increase the weight of positive sample. In XGB, the hyperparameter “scale_pos_weight” was set to 1 to adjust the imbalance of positive and negative samples.

In the medical field, logistic regression is extensively used to develop prediction models. In Elastic Net logistic regression, classifier was trained with both the L1 penalty and L2 penalty, and the hyperparameter “C” was set to 0.2 for constraining the model to avoid overfitting. In SVM, L1 regularisation constant was used to cut down the number of features and avoid overfitting, and the hyperparameter “C” was set to 0.1.

RF, GBM and XGB all use decision tree as the base learner [17]. RF uses an ensemble of independent decision trees, and the most likely outcome was determined by a majority vote [18]. In GBM, decision trees are constructed sequentially, and each new tree is fit to the residual error after the previous step [17]. XGB is a scalable end-to-end tree boosting system [16].

Considering that the model is easy to overfit with too many estimators or too deep tree depth, we controlled the number of estimator and tree depth to avoid overfitting. The RF classifier was trained with 80 estimators, and the maximum tree depth was constrained of 4. In GBM, learning rate was set at 0.01 to ensure the robustness of models. Classifier was trained using 100 estimators with a maximum tree depth of 2. XGB classifier was trained by 80 estimators with a maximum tree depth of 3, and the learning rate was set at 0.1.

All model hyperparameters were chosen via grid search five-fold cross-validation on the training set. Machine learning models were developed in Python 3.7.2 using the scikit-learn library.

Confidence interval (CI) was generated using block bootstrapping of the predictions in the test set. The test set was randomly sampled for 1000 times, and generated 1000 bootstrap samples. Performance metrics were calculated for each bootstrap sample, and then these metrics were sorted. The 95% CI was determined by the 25th and 975th values in the sorted list of metrics.

### Model comparison

To evaluate and compare different models, each model was applied to the test set to predict postoperative MACEs, and we drew a receiver operating characteristic curve (ROC) and a precision-recall curve (PRC) for each model. Area under the ROC (AUROC) is widely used to estimate the performance of binary classifiers. However, AUROC can generate misleading conclusions about model performance in condition of imbalanced data [11]. Area under the PRC (AUPRC) gives no credit for predicting true negatives. Compared with AUROC, it provides a more accurate interpretation of the model’s actual performance for classifier on imbalanced dataset [20]. In this study, we chose AUPRC as the main evaluation metric for model comparison.

Brier score is used to assess model calibration, which evaluate how close the risk estimated by the model is close to the observed probability. Lower Brier score value indicates better model performance. We calculated Brier score to evaluate the calibration of models.

Wilcoxon signed rank test was used to compare the value of AUROC, AUPRC and Brier score. The differences between values were considered to be statistically significant at the level of *p* < 0.05.

### Undersampling method

The current updated version of RCRI, issued by the Canadian Cardiovascular Society, was used to incorporate the B-type natriuretic peptide (BNP) measurement [21]. Compared with the original RCRI, this new version more accurately predicts the risk of postoperative MACEs [7]. We developed a rule based on the updated RCRI to evaluate patients’ risk of postoperative MACEs. Table 1 shows the details of this method. Anesthetists categorized patients into different risk bands during preoperative interviews. The clinicians paid more attention to patients in the high risk group, and conducted advance interventions to improve the patients’ physical condition. As a result, patients in the high risk group may have had better outcomes conversely. This phenomenon could influence the process of model development. We applied undersampling method to minimize this influence. We excluded patients in the high risk group who had no postoperative MACEs because their outcomes may have been influenced by clinical interventions. Then, we developed predictive model based on the undersampling set.

**Table 1:** Preoperative assessment rule of postoperative MACEs

Risk factor	point
History of ischaemic heart disease	1
History of congestive heart failure	1
History of cerebrovascular disease	1
Preoperative serum creatinine ≥ 177μMol/L	1
High risk surgery^a^	1
Insulin dependent diabetes mellitus	1
300 ng/L < BNP ≤ 6000 ng/L	1
6000 ng/L < BNP	2

In our hospital, anesthetists used this rule to estimate patient’s risk of postoperative MACEs during preoperative interview. Patients were divided into different risk bands according to following judgement criterion: Low risk: total point = 0; intermediate risk: 0 < total point < 3; high risk: total point ≥ 3. Abbreviations: *MACEs* Major adverse cardiovascular events, *BNP* B-type natriuretic peptide

^a^Major vascular surgery, cardiac surgery

The original test set included patients whose outcomes may have been influenced by clinical interventions, and this could impact the models’ performance. Thus, we excluded patients in the high risk group who had no postoperative MACEs from the original test set to form an undersampling test set for comparing the models’ performance.

We visualized variable importance to better understand the predictors’ influence on the model with the best performance. In order to simplify the model to ensure the ease of use in clinical settings, we excluded variables with little contribution to the best model. We compared performances of original model and reduced model to ensure the retainment of predictive ability.

## Results

### Patient characteristics

Of 5808 geriatric patients with surgery from June 25, 2019 to June 29, 2020, 103 patients were excluded, of whom 46 had active symptoms of MACEs before surgery, and 57 lost to follow-up. 5705 geriatric patients were enrolled in the final dataset. Supplementary table S2 shows details of patient characteristics. In total, 171(3.0%) patients developed postoperative MACEs within 30 days after surgery.

### Model comparison

**Fig. 1:**
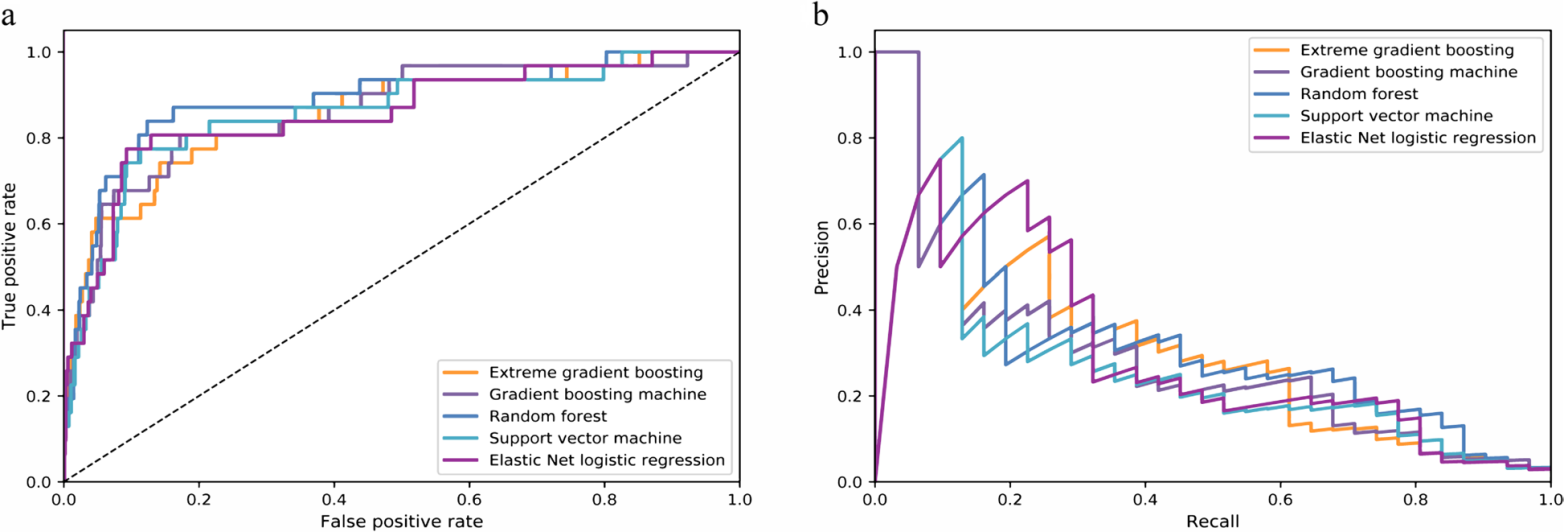
Performance characteristic curves of candidate models. (**a**) Receiver operating curves of each candidate model. (**b**) Precision-recall curves of each candidate model. This figure shows performance characteristic curves of candidate models trained by extreme gradient boosting, gradient boosting machine, random forest, support vector machine, and Elastic Net logistic regression

Figure 1 shows the ROCs and PRCs, respectively, of the models developed via different methods. All models achieved high AUROC values ranging from 0.856 (95%CI: 0.769–0.929) to 0.888 (95% CI: 0.804–0.951) (Table 2). The XGB model exhibited the greatest AUPRC (0.404[95% CI: 0.219–0.589]) and the lowest Brier score (0.024 [95% CI: 0.016–0.032]).

**Table 2:** Performance metrics of candidate models

Model	AUROC(95% CI)	AUPRC(95% CI)	Brier score(95% CI)
Extreme Gradient Boosting	0.870(0.786–0.938)	0.404(0.219–0.589)	0.024(0.016–0.032)
Gradient Boosting Machine	0.862(0.781–0.928)	0.287(0.133–0.431)	0.030(0.024–0.037)
Random forest	0.888(0.804–0.951)	0.305(0.151–0.481)	0.065(0.060–0.072)
Support vector machine	0.856(0.769–0.929)	0.247(0.111–0.414)	0.024(0.016–0.032)
Elastic Net logistic regression	0.857(0.775–0.925)	0.298(0.139–0.482)	0.105(0.079–0.139)

Performance metrics of models trained by extreme Gradient Boosting, Gradient Boosting Machine, random forest, support vector machine, and Elastic Net logistic regression. Abbreviations: *AUROC* Area under the receiver operating characteristic curve, *CI* Confidence interval, *AUPRC* Area under the precision-recall curve

### Comparison between models developed on original set and undersampling set

The outcomes of patients in the high risk group may have been influenced by clinical interventions. We applied undersampling method to minimize this influence (see the Methods above). 380 patients in the high risk group had no postoperative MACEs, and they were excluded from the original dataset. Supplementary table S3 shows details of patient characteristics in the undersampling dataset. Considering AUPRC and Brier score, the XGB model showed the best performance in the previous comparison between models based on different methods. Thus, we used XGB to develop model based on undersampling set. To compare the performance between the original model and the undersampling model, we applied the models to the undersampling test set, obtaining predictions of postoperative MACEs.

Compared with the model trained on the original set, the model trained on the undersampling set showed significantly higher values of AUROC(0.912[95% CI: 0.847–0.962] in undersampling model, 0.870[95% CI: 0.786–0.938] in original model, *p* < 0.001) and AUPRC(0.511[95% CI: 0.344–0.667] in undersampling model, 0.404[95% CI: 0.219–0.589] in original model, *p* < 0.001) (Table 3). For comparison of model calibration, the undersampling model had significantly lower Brier score(0.020 [ 95% CI: 0.013–0.028] in undersampling model, 0.024[95% CI: 0.016–0.032] in original model, *p* < 0.001).

**Table 3:** Performance of the original model compared with the undersampling model

Performance metric	Original model	Undersampling model	*p* value
AUROC(95% CI)	0.870(0.786–0.938)	0.912(0.847–0.962)	< 0.001
AUPRC(95% CI)	0.404(0.219–0.589)	0.511(0.344–0.667)	< 0.001
Brier score	0.024(0.016–0.032)	0.020(0.013–0.028)	< 0.001

Abbreviations: *AUROC* Area under the receiver operating characteristic curve, *CI* Confidence interval, *AUPRC* Area under the precision-recall curve

### Variable removal and feature importance

In order to simplify the model, we excluded 35 insignificant variables in XGB model, and these variables were also not important from clinical perspective. Compared with the undersampling model, the reduced undersampling model did not compromise the accuracy of risk prediction (AUPRC of 0.507[95% CI: 0.338–0.669] in the reduced undersampling model, AUPRC of 0.511[95% CI: 0.344–0.667] in the undersampling model, *p* = 0.36) (Table 4). For calibration, these models had the same Brier scores (0.020[95% CI: 0.013–0.028] for both, *p* = 0.20). Retaining all variables would increase the model complexity without meaningfully improvement of predictive ability, so we chose the reduced undersampling model to develop our calculating system. Supplementary table S4 shows the 84 variables included in the reduced undersampling model.

**Table 4:** Performance of the undersampling model compared with the reduced undersampling model

Performance metric	Undersampling model	Reduced Undersampling model	*p* value
AUROC(95% CI)	0.912(0.847–0.962)	0.896(0.826–0.953)	< 0.001
AUPRC(95% CI)	0.511(0.344–0.667)	0.507(0.338–0.669)	0.36
Brier score	0.020(0.013–0.028)	0.020(0.013–0.028)	0.20

Abbreviations: *AUROC* Area under the receiver operating characteristic curve, *CI* Confidence interval, *AUPRC* Area under the precision-recall curve

**Fig. 2:**
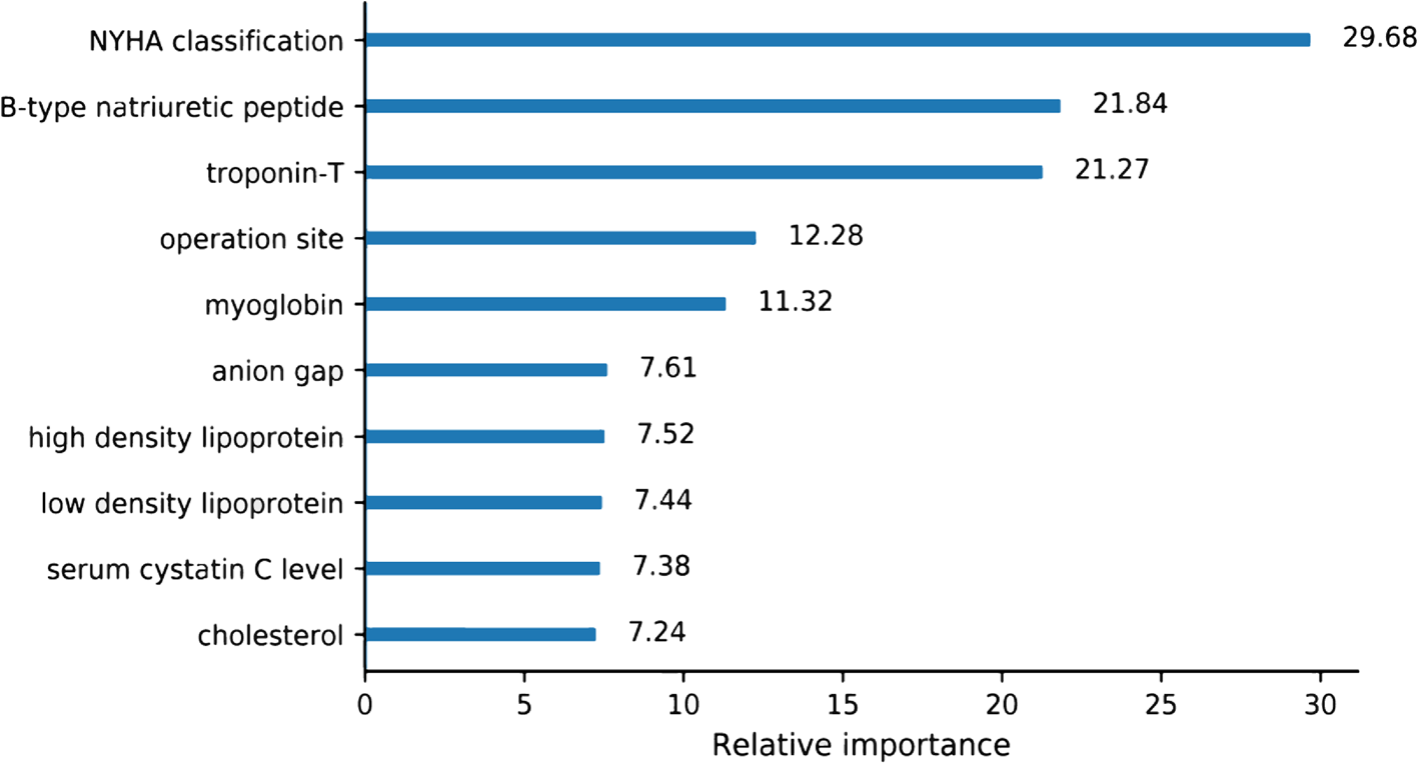
Importance matrix plot of the reduced undersampling XGB model. This figure shows the top ten important variables in reduced undersampling XGB model. Abbreviations: XGB: Extreme Gradient Boosting; NYHA: New York Heart Association

Top ten important variables in the reduced undersampling XGB model included New York Heart Association classification, BNP, troponin-T, operation site, myoglobin, anion gap, high density lipoprotein, low density lipoprotein, serum cystatin C level and cholesterol. (Fig. 2).

## Discussion

We conducted a prospective cohort study to develop machine learning models for preoperative prediction of postoperative MACEs in geriatric patients. The XGB model showed the best performance among these machine learning models. To minimize the influence of clinical intervention on patients’ outcomes, we used undersampling method according to the results of previous preoperative risk assessments. The model trained on the undersampling set showed improved performance. We excluded insignificant variables to ensure the ease of use in clinical settings, and the model retained equal predictive ability after removing insignificant variables. For convenient utilization in clinical practice, the model could be integrated into electronic medical records systems to ensure automatic data reading without the requirement of manual data input. Identifying patients with a high risk of postoperative MACEs prior to surgery can facilitate preoperative informed consent, perioperative management, and improvement of patients’ prognoses.

In other studies, the data of older and younger patients have often been pooled together. Considering that geriatric patients have age-related physiological specificities, ignoring age categories can cause inaccurate parameter estimation and may decrease the model’s discrimination ability in geriatric patients [9]. Current assessment tools developed on pooled data often underestimate the real cardiac risk in geriatric patients [9]. In this study, we specifically focused on the geriatric population to capture their particular characteristics.

Fritz and colleagues pointed out that clinicians were able to identify some abnormalities in patients and enacted interventions to improve their physiological conditions. Thus, patients with severe conditions may have better outcomes conversely to the expected result. This phenomenon could influence the process of model development [22]. To our knowledge, no previous study has determined any method to solve this problem. In our hospital, the anesthetists used the scale developed based on the updated RCRI to assess patients’ risk of postoperative MACEs during preoperative interviews. The clinicians paid more attention to patients in the high risk group and intervened in advance to improve their physical condition. To minimize this influence on patients’ outcomes, we applied undersampling method according to the results of preoperative risk assessments. The model trained on the undersampling training set showed higher predictive accuracy than model trained on the original dataset. The undersampling method successfully improved the model’s predictive ability.

Logistic regression imposes a linear and additive relationship between the predictors and the outcome, and this assumption might be incorrect considering the complex process underlying the development of postoperative MACEs [23]. In addition, multiple correlated features introduce noise in the process of model development using logistic regression, which may reduce predictive accuracy [24]. The nonlinear, nonparametric machine learning methods are capable of finding higher-dimensional interactions between features and developing predictive models with great accuracy [25, 26]. In our study, the high AUPRC and AUROC values achieved by machine learning methods are not commonly observed in other clinical predictive models [27, 28]. Additionally, machine learning techniques can be applied to imbalanced data and facilitate automation within electronic medical records systems [29].

In the present study, the XGB model showed the best performance. Previous studies which used different machine learning approaches to predict postoperative adverse events also achieved the best model performance through XGB [30, 31]. This evidence suggests that XGB might be more suitable than other machine learning methods for establishing predictive models of postoperative adverse events.

In this study, we predetermined risk factors and collected data prospectively. Most studies that develop predictive models are based on retrospective data [12, 13]. The factors incorporated in these models are restricted by data availability [32]. Certain predictors with potential prognostic implications may not be incorporated in retrospective study because of unavailability or incompleteness, and this limitation could be overcome through prospective study design [9].

Instead of simply dichotomizing comorbidities according to the presence or absence of each disease, we classified some diseases according to severity, which might improve the models’ predictive accuracy [33]. Laboratory tests objectively reflect patients’ present physiological condition and disease severity, so they have the potential advantages of predicting adverse events and guiding clinical decisions [34]. We included many laboratory tests in this study and regarded them as continuous variables instead of categorizing them according to thresholds. Categorization is biologically implausible because it would be unreasonable for a patient’s risk to change suddenly to either side of a threshold [35]. Preselecting cut points for continuous variables can cause information loss and decrease predictive accuracy [36].

Missing values are unavoidable in clinical practice. In our study, continues variables missing in fewer than 10% of cases were routinely collected during preoperative period, and the missing was likely to be random. These missing values were imputed using the k-nearest neighbor classification algorithm, which estimated missing values according to corresponding values of patients with similar condition. Some laboratory tests are known to be clinically associated with MACEs, but they are not routinely arranged to patients, like BNP and troponin-T. Clinicians often arrange these tests only for patients who are judged to be at high risk of postoperative MACEs, thus these variables often have high missing rates. In this study, we regarded missing values as a separate group for variables missing in more than 10% of cases instead of interpolating estimated values, which indicated that our model could classify patients without these measurements to a separate group. In this way, the model could learn the characteristics of patients who were deemed high risk for postoperative MACEs by clinicians. Improper imputation algorithms could influence the prediction performance [24]. We believe that our imputation algorithm is better than an arbitrary choice like mean imputation.

Some researchers regard machine learning as a “black box” and doubt its utility in clinical medicine [37]. We visualized the important variables in the optimal model to show some interpretability. Variables with great contribution to our model are also known to be associated with the development of MACEs from clinical perspective (such as BNP and troponin-T). In other hospitals, data are often collected and stored in different systems, and researchers need to integrate and harmonize data before using them to develop models [32]. We established a structured database of preoperative evaluation and postoperative follow-up in our hospital to ensure data integrity. Therefore, we can achieve continued data supply for further training and validation to improve the algorithm.

Our prediction model intends to serve as a supplement tool for perioperative cardiovascular risk management in geriatric patients. The model could be used to identify geriatric patients at high risk of postoperative MACEs, thus to guide anticipatory strategies, such as intraoperative invasive monitoring to ensure proper perfusion pressure and organ flow, and establishment of postoperative medical care plan, like intensive postoperative vital sign monitoring, arrangement of troponin or BNP measurement, and performance of postoperative electrocardiogram [38]. In addition, previous study demonstrated that probabilistic information is more accurately perceived by patients if presented as numbers, rather than words [39]. Our model could calculate individual probability of developing postoperative MACEs, thus to facilitate explicit communication with patients about the cardiovascular risk of surgery. Further research is necessary to quantify the benefit of this model in guiding interventions, reducing the incidence of postoperative MACEs, and improving patients’ outcomes.

Our study had several limitations. First, we used data from a single institution to develop and internally validate the predictive model. Future studies are needed to verify the generalizability of our model to other institutions. Second, this study covered all operation types. Subgroup analysis based on specific surgery type was not conducted because of the small number of patients in each group. The heterogeneity of different surgeries might represent a limitation of the model’s predictive ability in some subspecialties. However, the importance of our work lies in developing a predictive model available for widespread use instead of only for a specific type of surgery. Third, the low proportion of emergency cases and frail patients in our dataset limited the statistical power to identify emergency surgery and frailty as risk factors for postoperative MACEs. Frailty is a major factor in geriatric surgical outcomes [40], and emergency surgery is associated with postoperative pulmonary complications and acute kidney injury [41, 42]. But our model did not identify these variables as important predictors. We used the FRAIL Scale [43] to assess frailty in geriatric patients. The FRAIL Scale is appropriate for rapid bedside screening during preoperative interview, but it may not as accurate as other more complex scales, like the Robinson Frailty Score and Edmonton Frail Scale [44]. We may need to use other more accurate scales to assess frailty in further study. Considering the patient characteristics in our dataset, our prediction model may be more appropriate for geriatric patients with elective surgeries. Further studies are needed to explore whether emergency surgery and frailty are important risk factors for postoperative MACEs.

## Conclusions

In this prospective study, we used different machine learning methods to develop predictive models for preoperative prediction of postoperative MACEs in geriatric patients. The XGB model showed the best performance among these machine learning models. We applied undersampling method to minimize the influence of clinical intervention on patients’ outcomes, and this improved model performance. Our model could be integrated into electronic medical records systems and load related information automatically to calculate individualized predicted probabilities. Early identification of patients with high risk of postoperative MACEs could facilitate preoperative informed consent, early intervention, and allocation of medical resources.

## Supplementary Information

**Additional file 1:** **Supplementary Table S1**. Variables included in model development. **Supplementary Table S2**. Patient characteristics in the original set. **Supplementary Table S3**. Patient characteristics in the undersampling set. **Supplementary Table S4**. Variables included in the reduced undersampling model.

## Data Availability

The datasets used and analysed during the current study are available from the corresponding author on reasonable request.

## Abbreviations

MACEs: Major adverse cardiovascular events
RCRI: The revised cardiac risk index
MICA: The gupta myocardial infarction or cardiac arrest
XGB: Extreme gradient boosting
GBM: Gradient boosting machine
SVM: Support vector machine
CI: Confidence interval
ROC: Receiver operating characteristic curve
PRC: Precision-recall curve
AUROC: Area under the receiver operating characteristic curve
AUPRC: Area under the precision-recall curve
BNP: B-type natriuretic peptide
