# Supplementary material for: Machine learning prediction of postoperative major adverse cardiovascular events in geriatric patients: a prospective cohort study
Source: BMC Anesthesiol. 2022 Sep 10;22:284. doi: 10.1186/s12871-022-01827-x (PMC9463850; doi:10.1186/s12871-022-01827-x)
Supplement: Supplementary file 1 — Additional file 1: Supplementary Table S1. Variables included in model development. Supplementary Table S2. Patient characteristics in the original set. Supplementary Table S3. Patient characteristics in the undersampling set. Supplementary Table S4. Variables included in the reduced undersampling model. [file 12871_2022_1827_MOESM1_ESM.docx]

**Machine learning prediction of postoperative major adverse cardiovascular events in geriatric patients: a prospective cohort study -Supplementary data**

Supplementary Table S1. Variables included in model development.

| Variable | Missing rate |
| --- | --- |
| Demographic |  |
| age | 0.00% |
| sex | 0.00% |
| weight | 0.90% |
| height | 1.21% |
| body mass index | 1.32% |
| allergic history | 0.00% |
| smoking | 0.00% |
| alcohol | 0.00% |
| Preoperative vital signs |  |
| body temperature | 0.73% |
| heart rate | 0.24% |
| respiratory rate | 0.17% |
| systolic blood pressure | 0.39% |
| diastolic blood pressure | 0.28% |
| Laboratory tests |  |
| RDW-CV | 2.77% |
| RDW-SD | 2.81% |
| Mean corpuscular hemoglobin | 2.66% |
| Mean corpuscular hemoglobin concentration | 2.66% |
| Mean corpuscular volume | 2.66% |
| white blood cell count | 2.68% |
| red blood cell count | 2.66% |
| Blood platelet count | 2.72% |
| hemoglobin | 2.66% |
| hematocrit | 2.40% |
| basophil percentage | 2.90% |
| absolute basophil count | 2.88% |
| absolute acidophil count | 2.77% |
| acidophil percentage | 2.77% |
| lymphocyte percentage | 2.68% |
| absolute lymphocyte count | 2.70% |
| neutrophil granulocyte percentage | 2.68% |
| absolute neutrophil granulocyte count | 2.70% |
| monocyte percentage | 2.68% |
| absolute monocyte count | 2.68% |
| Prothrombin time | 8.16% |
| Activated partial thromboplastin time | 8.20% |
| fibrinogen | 8.22% |
| Thrombin time | 8.20% |
| International normalized ratio | 8.16% |
| ALT | 2.48% |
| AST | 2.48% |
| AST/ALT | 2.48% |
| blood urea | 2.42% |
| uric acid | 2.42% |
| total bilirubin | 2.48% |
| direct bilirubin | 2.48% |
| indirect bilirubin | 2.48% |
| total protein | 2.48% |
| globulin | 2.48% |
| albumin | 2.48% |
| albumin-globulin ratio | 2.48% |
| Alkaline phosphatase | 2.48% |
| creatinine | 2.42% |
| blood glucose | 2.49% |
| serum cystatin C level | 2.44% |
| Glutamyl transpeptidase | 2.48% |
| serum sodium | 2.53% |
| serum chlorine | 2.53% |
| serum phosphorus | 2.92% |
| serum magnesium | 2.92% |
| serum potassium | 2.53% |
| serum calcium | 2.92% |
| SpO_2_ | 1.61% |
| CO_2_CP | 2.97% |
| blood β-hydroxybutyrate | 3.87% |
| hydroxybutyrate dehydrogenase | 2.77% |
| B-type natriuretic peptide | 81.91% |
| creatine kinase-MB isoenzyme | 79.07% |
| myoglobin | 79.07% |
| troponin-T | 77.97% |
| Creatine kinase | 2.77% |
| Lactate dehydrogenase | 2.75% |
| Anion gap | 2.81% |
| total bile acid | 2.84% |
| eGFR | 2.83% |
| cholesterol | 2.72% |
| low density lipoprotein | 2.72% |
| high density lipoprotein | 2.72% |
| triglyceride | 2.72% |
| Comorbidities |  |
| hypertension | 0.00% |
| ischemic heart disease | 0.00% |
| congestive heart failure | 0.02% |
| arrhythmia | 0.00% |
| respiratory infection within last 1 month | 0.12% |
| COPD | 0.00% |
| pleural effusion | 0.00% |
| pneumothorax | 0.00% |
| stroke | 0.00% |
| psychiatric disorder | 0.00% |
| neuromuscular disease | 0.00% |
| diabetes mellitus | 0.00% |
| pheochromocytoma | 0.00% |
| liver disease | 0.00% |
| kidney disease | 0.00% |
| digestive tract hemorrhage within last 1 week | 0.00% |
| gastroesophageal reflux | 0.00% |
| peptic ulcer | 0.00% |
| coagulation disorders | 0.00% |
| hematological disease | 0.00% |
| immunity dysfunction | 0.00% |
| autoimmune disease | 0.00% |
| sleep apnea syndrome | 0.00% |
| airway obstruction | 0.00% |
| dyspnea | 0.00% |
| difficult ventilation history | 0.00% |
| difficult intubation history | 0.00% |
| Surgical details |  |
| emergency surgery | 0.00% |
| operation site | 0.00% |
| operation type | 0.00% |
| estimated operation time | 0.00% |
| estimated blood loss | 0.00% |
| Other variables |  |
| the FRAIL Scale | 0.00% |
| ASA classification | 0.00% |
| general condition | 0.13% |
| NYHA classification | 1.09% |
| daily activity ability | 1.73% |
| ventilation modes | 0.00% |
| breath-holding test | 1.55% |
| conscious state | 0.09% |
| Mallampati airway classification | 0.54% |
| mandibular protrusion test | 0.00% |
| limited mouth opening | 0.00% |

Footnote: Abbreviations: RDW-CV: Coefficient of variation of red blood cell distribution width; RDW-SD: Standard deviation of red blood cell distribution width; ALT: Alanine aminotransferase; AST: Aspartate aminotransferase; SpO_2_: Oxygen saturation; CO_2_CP: Carbon dioxide combining power; eGFR: Estimated glomerular filtration rate; COPD: Chronic obstructive pulmonary disease; ASA: American Society of Anesthesiologists; NYHA: New York Heart Association.

Supplementary Table S2. Patient characteristics in the original set.

| Variable | Training set (n=4564) | Test set  (n=1141) | *p* Value |
| --- | --- | --- | --- |
| male | 2575(56.42) | 655(57.41) | 0.57 |
| age | 72.01±5.77 | 72.15±5.68 | 0.14 |
| height | 160.39±8.04 | 160.13±8.04 | 0.25 |
| weight | 60.57±10.52 | 60.49±9.50 | 0.49 |
| BMI | 23.52±3.47 | 23.59±3.38 | 0.38 |
| smoking | 336(7.36) | 88(7.71) | 0.73 |
| alcohol | 52(1.14) | 15(1.31) | 0.74 |
| allergic history | 467(10.23) | 127(11.13) | 0.40 |
| systolic blood pressure | 134.52±17.65 | 135.25±17.90 | 0.11 |
| diastolic blood pressure | 79.69±11.68 | 79.67±11.41 | 0.45 |
| respiratory rate | 19.56±2.21 | 19.57±2.96 | 0.16 |
| heart rate | 79.56±12.92 | 79.13±12.31 | 0.16 |
| body temperature | 36.46±0.27 | 36.47±0.25 | 0.20 |
| hypertension |  |  | 0.43 |
| I | 540(11.83) | 143(12.53) |  |
| II | 699(15.32) | 175(15.34) |  |
| III | 403(8.83) | 116(10.17) |  |
| ischemic heart disease |  |  | 0.83 |
| mild stenosis | 63(1.38) | 13(1.14) |  |
| moderate stenosis | 28(0.61) | 7(0.61) |  |
| severe stenosis | 23(0.50) | 4(0.35) |  |
| congestive heart failure | 15(0.33) | 6(0.53) | 0.48 |
| arrhythmia |  |  | 0.97 |
| low risk | 184(4.03) | 43(3.77) |  |
| medium risk | 104(2.28) | 25(2.19) |  |
| high risk | 47(1.03) | 11(0.96) |  |
| dyspnea |  |  | 0.35 |
| I | 130(2.85) | 43(3.77) |  |
| II | 229(5.02) | 59(5.17) |  |
| III | 63(1.38) | 14(1.23) |  |
| IV | 20(0.44) | 2(0.18) |  |
| respiratory infection within last 1 month | 86(1.88) | 22(1.93) | 0.98 |
| COPD |  |  | 0.21 |
| stable | 194(4.25) | 57(5.00) |  |
| exacerbations | 7(0.15) | 0(0.00) |  |
| repeated exacerbations within 1 year≥3 | 5(0.11) | 3(0.26) |  |
| pneumothorax | 2(0.04) | 2(0.18) | 0.38 |
| pleural effusion | 6(0.13) | 2(0.18) | 0.93 |
| stroke |  |  | 0.68 |
| >12 months | 66(1.45) | 15(1.31) |  |
| 6~12 months | 9(0.20) | 1(0.09) |  |
| 1~6 months | 7(0.15) | 3(0.26) |  |
| <1 month | 9(0.20) | 4(0.35) |  |
| psychiatric disorder | 30(0.66) | 9(0.79) | 0.78 |
| neuromuscular disease | 28(0.61) | 9(0.79) | 0.65 |
| diabetes mellitus |  |  | 0.20 |
| no intervention | 34(0.74) | 9(0.79) |  |
| diet therapy | 31(0.68) | 9(0.79) |  |
| oral hypoglycaemic therapy | 300(6.57) | 96(8.41) |  |
| insulin dependent | 148(3.24) | 30(2.63) |  |
| pheochromocytoma | 9(0.20) | 3(0.26) | 0.94 |
| liver disease | 138(3.02) | 29(2.54) | 0.44 |
| kidney disease | 44(0.96) | 10(0.88) | 0.92 |
| digestive tract hemorrhage within last 1 week | 36(0.79) | 10(0.88) | 0.91 |
| gastroesophageal reflux | 57(1.25) | 9(0.79) | 0.25 |
| peptic ulcer | 52(1.14) | 10(0.88) | 0.54 |
| coagulation disorders | 8(0.18) | 0(0.00) | 0.33 |
| hematological disease | 12(0.26) | 1(0.09) | 0.45 |
| immunity dysfunction | 1(0.02) | 1(0.09) | 0.86 |
| autoimmune disease | 7(0.15) | 3(0.26) | 0.69 |
| sleep apnea syndrome | 428(9.38) | 104(9.11) | 0.83 |
| airway obstruction | 50(1.10) | 11(0.96) | 0.82 |
| difficult ventilation history |  |  | 0.15 |
| suspected | 20(0.44) | 10(0.88) |  |
| yes | 8(0.18) | 1(0.09) |  |
| difficult intubation history |  |  | 0.30 |
| suspected | 12(0.26) | 5(0.44) |  |
| yes | 6(0.13) | 0(0.00) |  |
| the FRAIL Scale |  |  | 0.13 |
| fit | 4437(97.22) | 1121(98.25) |  |
| pre-frail | 82(1.80) | 14(1.23) |  |
| frail | 45(0.99) | 6(0.53) |  |
| emergency surgery | 24(0.53) | 8(0.70) | 0.63 |
| operation type |  |  |  |
| open surgery | 2667(58.44) | 674(59.07) | 0.72 |
| endoscopic surgery | 1918(42.02) | 471(41.28) | 0.67 |
| estimated operation time |  |  | 0.84 |
| <2h | 2397(52.52) | 591(51.80) |  |
| 2~4h | 1745(38.23) | 447(39.18) |  |
| ≥4h | 422(9.25) | 103(9.03) |  |
| estimated blood loss |  |  | 0.39 |
| <10% estimated blood volume | 3951(86.57) | 1005(88.08) |  |
| 10~25% estimated blood volume | 583(12.77) | 130(11.39) |  |
| >25% estimated blood volume | 30(0.66) | 6(0.53) |  |
| operation site |  |  | 0.78 |
| orthopaedics | 880(19.28) | 216(18.93) |  |
| general surgery | 2529(55.41) | 619(54.25) |  |
| thoracic surgery | 398(8.72) | 109(9.55) |  |
| cardiovascular surgery | 207(4.54) | 49(4.29) |  |
| other | 550(12.05) | 148(12.97) |  |
| ASA classification |  |  | 0.26 |
| I | 12(0.26) | 3(0.26) |  |
| II | 2227(48.79) | 560(49.08) |  |
| III | 2298(50.35) | 565(49.52) |  |
| IV | 27(0.59) | 13(1.14) |  |
| conscious state |  |  | 0.24 |
| sober | 4551(99.72) | 1137(99.65) |  |
| somnolence | 11(0.24) | 2(0.18) |  |
| lethargy | 1(0.02) | 0(0.00) |  |
| light coma | 1(0.02) | 1(0.09) |  |
| deep coma | 0(0.00) | 1(0.09) |  |
| general condition |  |  | 0.45 |
| partial dependence | 3019(66.15) | 773(67.75) |  |
| dependence | 1449(31.75) | 349(30.59) |  |
| NYHA classification |  |  | 0.61 |
| I | 2492(54.60) | 622(54.51) |  |
| II | 1682(36.85) | 430(37.69) |  |
| III | 254(5.57) | 54(4.73) |  |
| IV | 17(0.37) | 2(0.18) |  |
| daily activity ability |  |  | 0.92 |
| >6MET | 883(19.35) | 219(19.19) |  |
| 3-6MET | 2672(58.55) | 675(59.16) |  |
| <3MET | 1009(22.11) | 247(21.65) |  |
| ventilation modes |  |  | 0.81 |
| spontaneous respiration | 4539(99.45) | 1135(99.47) |  |
| assisted ventilation | 13(0.28) | 4(0.35) |  |
| controlled ventilation | 12(0.26) | 2(0.18) |  |
| breath-holding test |  |  | 0.63 |
| ≥30s | 1800(39.44) | 427(37.42) |  |
| 20~29s | 2325(50.94) | 605(53.02) |  |
| 10~19s | 407(8.92) | 101(8.85) |  |
| <10s | 32(0.70) | 8(0.70) |  |
| limited mouth opening | 138(3.02) | 37(3.24) | 0.77 |
| Mallampati airway classification |  |  | 0.87 |
| I | 911(19.96) | 226(19.81) |  |
| II | 3157(69.17) | 798(69.94) |  |
| III | 454(9.95) | 109(9.55) |  |
| IV | 42(0.92) | 8(0.7) |  |
| mandibular protrusion test | 40(0.88) | 14(1.23) | 0.36 |

Footnote: Patient characteristics for the cohort used for training and testing models in the original set. For continuous variables, data is presented in means and standard deviations. For categorical variables, data is presented in number of patients and percent of the cohort. Abbreviations: BMI: Body Mass Index; NYHA: New York Heart Association; MET: Metabolic equivalent; COPD: Chronic obstructive pulmonary disease; ASA: American Society of Anesthesiologists.

Supplementary Table S3. Patient characteristics in the undersampling set.

| Variable | Training set (n=4251) | Test set  (n=1074) | *p* Value |
| --- | --- | --- | --- |
| male | 2392(56.27) | 617(57.45) | 0.51 |
| age | 71.75±5.51 | 71.86±5.45 | 0.21 |
| height | 160.45±8.01 | 160.19±80 | 0.30 |
| weight | 60.67±10.46 | 60.55±9.42 | 0.45 |
| BMI | 23.54±3.44 | 23.6±3.41 | 0.46 |
| smoking | 310(7.29) | 83(7.73) | 0.67 |
| alcohol | 50(1.18) | 15(1.4) | 0.67 |
| allergic history | 436(10.26) | 119(11.08) | 0.46 |
| systolic blood pressure | 134.49±17.48 | 135.07±17.60 | 0.17 |
| diastolic blood pressure | 79.91±11.58 | 79.74±11.07 | 0.28 |
| respiratory rate | 19.53±2.02 | 19.56±3.03 | 0.18 |
| heart rate | 79.47±12.77 | 79.12±12.12 | 0.20 |
| body temperature | 36.46±0.27 | 36.46±0.25 | 0.25 |
| hypertension |  |  | 0.39 |
| I | 500(11.76) | 131(12.20) |  |
| II | 640(15.06) | 158(14.71) |  |
| III | 353(8.30) | 106(9.87) |  |
| ischemic heart disease |  |  | 0.75 |
| mild stenosis | 52(1.22) | 10(0.93) |  |
| moderate stenosis | 23(0.54) | 6(0.56) |  |
| severe stenosis | 14(0.33) | 2(0.19) |  |
| congestive heart failure | 9(0.21) | 4(0.37) | 0.54 |
| arrhythmia |  |  | 0.99 |
| low risk | 161(3.79) | 40(3.72) |  |
| medium risk | 80(1.88) | 19(1.77) |  |
| high risk | 13(0.31) | 3(0.28) |  |
| dyspnea |  |  | 0.40 |
| I | 122(2.87) | 40(3.72) |  |
| II | 196(4.61) | 51(4.75) |  |
| III | 28(0.66) | 8(0.74) |  |
| IV | 7(0.16) | 0(0.00) |  |
| respiratory infection within last 1 month | 68(1.60) | 19(1.77) | 0.80 |
| COPD |  |  | 0.28 |
| stable | 160(3.76) | 46(4.28) |  |
| exacerbations | 4(0.09) | 0(0.00) |  |
| repeated exacerbations within 1 year≥3 | 2(0.05) | 2(0.19) |  |
| pneumothorax | 2(0.05) | 2(0.19) | 0.39 |
| pleural effusion | 6(0.14) | 2(0.19) | 0.92 |
| stroke |  |  | 0.88 |
| >12 months | 51(1.2) | 15(1.40) |  |
| 6~12 months | 7(0.16) | 1(0.09) |  |
| 1~6 months | 4(0.09) | 1(0.09) |  |
| <1 month | 7(0.16) | 3(0.28) |  |
| psychiatric disorder | 24(0.56) | 8(0.74) | 0.64 |
| neuromuscular disease | 26(0.61) | 8(0.74) | 0.78 |
| diabetes mellitus |  |  | 0.13 |
| no intervention | 30(0.71) | 9(0.84) |  |
| diet therapy | 31(0.73) | 9(0.84) |  |
| oral hypoglycaemic therapy | 276(6.49) | 92(8.57) |  |
| insulin dependent | 133(3.13) | 27(2.51) |  |
| pheochromocytoma | 9(0.21) | 3(0.28) | 0.95 |
| liver disease | 129(3.03) | 29(2.70) | 0.63 |
| kidney disease | 41(0.96) | 9(0.84) | 0.84 |
| digestive tract hemorrhage within last 1 week | 31(0.73) | 9(0.84) | 0.86 |
| gastroesophageal reflux | 54(1.27) | 9(0.84) | 0.31 |
| peptic ulcer | 49(1.15) | 10(0.93) | 0.65 |
| coagulation disorders | 8(0.19) | 0(0.00) | 0.33 |
| hematological disease | 12(0.28) | 1(0.09) | 0.44 |
| immunity dysfunction | 1(0.02) | 1(0.09) | 0.86 |
| autoimmune disease | 4(0.09) | 3(0.28) | 0.31 |
| sleep apnea syndrome | 398(9.36) | 94(8.75) | 0.58 |
| airway obstruction | 46(1.08) | 11(1.02) | 1.00 |
| difficult ventilation history |  |  | 0.24 |
| suspected | 16(0.38) | 8(0.74) |  |
| yes | 7(0.16) | 1(0.09) |  |
| difficult intubation history |  |  | 0.32 |
| suspected | 8(0.19) | 4(0.37) |  |
| yes | 4(0.09) | 0(0.00) |  |
| the FRAIL Scale |  |  | 0.17 |
| fit | 4174(98.19) | 1063(98.98) |  |
| pre-frail | 56(1.32) | 9(0.84) |  |
| frail | 21(0.49) | 2(0.19) |  |
| emergency surgery | 21(0.49) | 6(0.56) | 0.98 |
| operation type |  |  |  |
| open surgery | 2463(57.94) | 630(58.66) | 0.69 |
| endoscopic surgery | 1810(42.58) | 452(42.09) | 0.80 |
| estimated operation time |  |  | 0.65 |
| <2h | 2246(52.83) | 557(51.86) |  |
| 2~4h | 1624(38.2) | 426(39.66) |  |
| ≥4h | 381(8.96) | 91(8.47) |  |
| estimated blood loss |  |  | 0.23 |
| <10% estimated blood volume | 3710(87.27) | 958(89.2) |  |
| 10~25% estimated blood volume | 517(12.16) | 111(10.34) |  |
| >25% estimated blood volume | 24(0.56) | 5(0.47) |  |
| operation site |  |  | 0.72 |
| orthopaedics | 809(19.03) | 196(18.25) |  |
| general surgery | 2391(56.25) | 592(55.12) |  |
| thoracic surgery | 379(8.92) | 105(9.78) |  |
| cardiovascular surgery | 139(3.27) | 34(3.17) |  |
| other | 533(12.54) | 147(13.69) |  |
| ASA classification |  |  | 0.16 |
| I | 10(0.24) | 3(0.28) |  |
| II | 2188(51.47) | 549(51.12) |  |
| III | 2039(47.97) | 513(47.77) |  |
| IV | 14(0.33) | 9(0.84) |  |
| conscious state |  |  | 0.04 |
| sober | 4245(99.86) | 1071(99.72) |  |
| somnolence | 6(0.14) | 1(0.09) |  |
| light coma | 0(0.00) | 1(0.09) |  |
| deep coma | 0(0.00) | 1(0.09) |  |
| general condition |  |  | 0.57 |
| partial dependence | 2932(68.97) | 751(69.93) |  |
| dependence | 1269(29.85) | 314(29.24) |  |
| NYHA classification |  |  | 0.66 |
| I | 2461(57.89) | 617(57.45) |  |
| II | 1629(38.32) | 419(39.01) |  |
| III | 39(0.92) | 7(0.65) |  |
| IV | 6(0.14) | 0(0.00) |  |
| daily activity ability |  |  | 0.93 |
| >6MET | 857(20.16) | 212(19.74) |  |
| 3-6MET | 2586(60.83) | 660(61.45) |  |
| <3MET | 808(19.01) | 202(18.81) |  |
| ventilation modes |  |  | 0.74 |
| spontaneous respiration | 4232(99.55) | 1071(99.72) |  |
| assisted ventilation | 7(0.16) | 1(0.09) |  |
| controlled ventilation | 12(0.28) | 2(0.19) |  |
| breath-holding test |  |  | 0.32 |
| ≥30s | 1727(40.63) | 407(37.90) |  |
| 20~29s | 2179(51.26) | 576(53.63) |  |
| 10~19s | 330(7.76) | 85(7.91) |  |
| <10s | 15(0.35) | 6(0.56) |  |
| limited mouth opening | 124(2.92) | 36(3.35) |  |
| Mallampati airway classification |  |  | 0.93 |
| I | 871(20.49) | 216(20.11) |  |
| II | 2944(69.25) | 751(69.93) |  |
| III | 401(9.43) | 100(9.31) |  |
| IV | 35(0.82) | 7(0.65) |  |
| mandibular protrusion test | 36(0.85) | 12(1.12) | 0.51 |

Footnote: Patient characteristics for the cohort used for training and testing models in the undersampling set. For continuous variables, data is presented in means and standard deviations. For categorical variables, data is presented in number of patients and percent of the cohort. Abbreviations: BMI: Body Mass Index; NYHA: New York Heart Association; MET: Metabolic equivalent; COPD: Chronic obstructive pulmonary disease; ASA: American Society of Anesthesiologists.

Supplementary Table S4. Variables included in the reduced undersampling model.

| Variable group | Variables |
| --- | --- |
| Demographic | age, weight, height, body mass index, smoking, alcohol |
| Preoperative vital signs | heart rate, systolic blood pressure, diastolic blood pressure |
| Laboratory tests | Blood routine: RDW-SD, MCH, MCHC, MCV, white blood cell count, red blood cell count, PLT, hemoglobin, hematocrit, basophil percentage, absolute basophil count, absolute acidophil count, acidophil percentage, lymphocyte percentage, absolute lymphocyte count, absolute neutrophil granulocyte count, monocyte percentage, absolute monocyte count; coagulation tests: PT, APTT, fibrinogen, TT, INR; blood biochemistry: ALT, AST, AST/ALT, blood urea, uric acid, total bilirubin, direct bilirubin, indirect bilirubin, total protein, globulin, albumin, albumin-globulin ratio, ALP, creatinine, blood glucose, serum cystatin C level, GGT, serum sodium, serum chlorine, serum magnesium, serum potassium, serum calcium; arterial blood gas: SpO_2_, CO_2_CP; Other laboratory tests: hydroxybutyrate dehydrogenase, B-type natriuretic peptide, creatine kinase-MB isoenzyme, myoglobin, troponin-T, CK, LDH, AG, total bile acid, eGFR, cholesterol, low density lipoprotein, high density lipoprotein, triglyceride |
| Comorbidities | hypertension, ischemic heart disease, congestive heart failure, arrhythmia, stroke, diabetes mellitus |
| Surgical details | operation site, operation type, estimated operation time |
| Other variables | ASA classification, general condition, NYHA classification, daily activity ability, breath-holding test |

Footnote: Abbreviations: RDW-SD: Standard deviation of red blood cell distribution width; MCH: Mean corpuscular hemoglobin; MCHC: Mean corpuscular hemoglobin concentration; MCV: Mean corpuscular volume; PLT: Blood platelet count; PT: Prothrombin time; APTT: Activated partial thromboplastin time; TT: Thrombin time; INR: International normalized ratio; ALT: Alanine aminotransferase; AST: Aspartate aminotransferase; ALP: Alkaline phosphatase; GGT: Glutamyl transpeptidase; SpO_2_: Oxygen saturation; CO_2_CP: Carbon dioxide combining power; CK: Creatine kinase; LDH: Lactate dehydrogenase; AG: Anion gap; eGFR: Estimated glomerular filtration rate; ASA: American Society of Anesthesiologists; NYHA: New York Heart Association.
